# Early Partitioning of Structural Paralog Diversity Shapes Immune Evolution Across Habitat Transitions in Gobiiform Fishes

**DOI:** 10.64898/2026.07.27.740993

**Published:** 2026-07-29

**Authors:** Katerina L. Zapfe, Ian Birchler De Allende, Aditt Mahadik, Gerard R. Nasser, Bruno Frédérich, Jeffrey A. Yoder, Alex Dornburg

## Abstract

Habitat transitions expose species lineages to novel pathogen regimes and are often hypothesized to drive adaptive diversification of immune gene families. However, the temporal association between gene family diversification and ecological change remains unresolved. To gain deeper insight into the relationship between the diversification of species and the evolution of their immune system, we investigated the evolutionary history of Toll-like receptors (TLRs) across Gobiiformes, a clade characterized by repeated transitions across aquatic and amphibious environments. TLRs are well-studied membrane-bound pattern recognition receptors that play crucial roles in detecting pathogens and immune activation. Phylogenomic, structural, and sequence analyses reveal that a major expansion of TLR22 predates many ecological transitions, with early paralog diversification partitioning receptor architectures into distinct structural regimes that persist across lineages. Subsequent evolution is concentrated in the extracellular ligand-binding, leucine-rich repeat (LRR) domains, where localized sequence and structural variation enables likely functional tuning without major architectural innovation. These results indicate that ecological transitions do not require repeated evolution of new immune receptor forms, but can instead be facilitated by reconfiguration of pre-existing immunogenetic diversity. These findings raise the possibility that expansions of immune gene families occurring early in a clade’s evolutionary history may commonly persist and subsequently act as substrates for evolutionary responses to environmental change.

## Full Text Availability

The license terms selected by the author(s) for this preprint version do not permit archiving in PMC. The full text is available from the preprint server.

